# Increased methotrexate intolerance in juvenile idiopathic arthritis compared to acute lymphoblastic leukaemia in children

**DOI:** 10.1371/journal.pone.0219539

**Published:** 2019-07-11

**Authors:** Nini Kyvsgaard, Torben Stamm Mikkelsen, Mikael Thastum, Anne Estmann Christensen, Peder Skov Wehner, Karsten Nysom, Troels Herlin

**Affiliations:** 1 Paediatric and Adolescent Medicine, Department of Clinical Medicine, Aarhus University Hospital, Aarhus, Denmark; 2 Department of Psychology and Behavioural Sciences, Aarhus BSS, Aarhus University, Aarhus, Denmark; 3 Department of Paediatric Rheumatology, H.C. Andersen Children’s Hospital, Odense University Hospital, Odense, Denmark; 4 Department of Pediatric Hematology and Oncology, H.C. Andersen Children’s Hospital, Odense University Hospital, Odense, Denmark; 5 Pediatrics and Adolescent Medicine, Rigshospitalet, Copenhagen, Denmark; Cleveland Clinic, UNITED STATES

## Abstract

**Objectives:**

To analyse the internal consistency of an adaption of the methotrexate intolerance severity score (MISS); and to describe and compare the level of methotrexate intolerance evaluated by the MISS in Danish children with juvenile idiopathic arthritis (JIA) or acute lymphoblastic leukaemia (ALL), treated with low-dose methotrexate (MTX).

**Methods:**

Cross-sectional study of children diagnosed with JIA or ALL, treated with low-dose MTX, aged 9 years or above, and cognitively intact. The patient’s parents completed the MISS. MTX intolerance was defined as a total MISS score above 6.

**Results:**

We enrolled 120 children with JIA and 23 children with ALL. The MISS had a good internal consistency in the JIA group. The median MISS score was higher in the JIA group than in the ALL group (JIA: 8; ALL: 1; p<0.0001); and the JIA group had a larger proportion of MTX intolerant children than the ALL group (JIA: 73/120; ALL: 4/23; p<0.001). Within both the JIA group and the ALL group, the MISS total score was not significantly correlated with age, MTX dose or the duration of low-dose MTX treatment.

**Conclusion:**

In the JIA group the level of MTX intolerance was higher and more attributed to anticipatory, associative and behavioural symptoms than in the ALL group. The MISS may help to uncover whether MTX intolerance is present and which aspects are affected in the individual patient, thus guiding intervention. The MISS may also be applicable within leukaemia care.

## Introduction

Low-dose methotrexate (MTX) is a cornerstone in the treatment of juvenile idiopathic arthritis (JIA) with polyarticular involvement [1–3] and in the maintenance treatment of acute lymphoblastic leukaemia (ALL) in combination with 6-mercaptopurine [4].

Gastrointestinal adverse effects, including anticipatory and associative symptoms, are described to be a significant clinical challenge to low-dose MTX treatment in JIA [5–7]. With the increasing survival rates of children with ALL [8–10] the role of adverse effects related to the maintenance treatment has gained higher significance, but there are few studies focusing on low-dose MTX.

Tools quantifying gastrointestinal adverse effects to low-dose MTX are crucial for identification and monitoring affected patients in order to assist patients in maintaining a superior health-related quality of life [11] and adhering to therapy. The methotrexate intolerance severity score (MISS) has been developed in a Dutch JIA cohort as a quantitative measure of specifically the low-dose MTX-associated gastrointestinal adverse effects and also incorporates anticipatory, associative, and behavioural complaints [6].

MTX intolerance appears more common among children with JIA compared to children with leukaemia based on clinical experience, but to our knowledge no previous studies have specifically investigated and compared MTX intolerance between JIA and childhood leukaemia. Important lessons may be learnt regarding MTX intolerance through the analysis and comparison of two disease entities using the same tool.

Our objectives were to analyse the internal consistency of a Danish version of the MISS; and to describe MTX intolerance, evaluated by the MISS, in a group of children with JIA and a group of children with ALL.

## Methods

### Study design

The study was cross-sectional. Participants and their families were only seen once. On the day of enrolment the patients’ parents completed the MISS, the families were asked two questions about MTX treatment compliance, and a senior physician completed a global assessment of the patient’s current level of MTX intolerance (PGA_MTX_).

### Ethics

The study was registered on clinicaltrials.gov (NCT02528435), performed in accordance with the Declaration of Helsinki and approved by the local research ethics committee (Central Denmark Region) (M-20110160) and the Danish Data Protection Agency (1-16-02-429-15). Prior to enrolment, all participating families provided written informed consent.

### Study population

The study population consisted of children either diagnosed with JIA according to the International League of Associations for Rheumatology criteria [12] or diagnosed with ALL and treated with low-dose MTX and 6-mercatopurine as part of the maintenance treatment on the Nordic Society of Paediatric Haematology and Oncology (NOPHO) ALL-2008 protocol [4,13]. All children were followed at our paediatric rheumatology or oncology outpatient clinics and were eligible if currently treated with low-dose MTX and aged 9 years or above. Children were excluded if they were cognitively impaired or not fluent in Danish. Children with JIA were enrolled from December 2013 until July 2016; children with ALL were enrolled from April 2015 until August 2017.

For every patient a case report form (CRF) was completed using REDCap (Research Electronic Data Capture) hosted at the Department of Clinical Medicine, Aarhus University [14]. The CRF contained information on the patients’ gender, age at enrolment, dose of MTX (mg/m^2^/week) and the route of administration (oral or subcutaneous) of MTX at enrolment, and whether they at a time prior to enrolment had received low-dose MTX through the alternate route of administration. Furthermore, whether folic acid and antiemetic medicine were prescribed at enrolment. The duration of low-dose MTX treatment prior to enrolment in this study was calculated from the date of treatment initiation to the date of enrolment. For the children with ALL, the date of treatment initiation was the date the children commenced the maintenance treatment with low-dose MTX and 6-mercaptopurine (after completed re-inductions), in accordance with their designated risk group determined by the NOPHO ALL-2008 protocol: the high risk group at treatment week 105, the intermediate risk group at treatment week 66 and the standard risk group at treatment week 58 [13]. For children with JIA who had re-commenced treatment with MTX due to a disease relapse (after a MTX treatment cessation) the most recent commencement date was selected.

### MTX intolerance

The MISS was developed in an electronic version developed using Survey Xact software (Ramboll). The parents were instructed to focus on their child’s current status of MTX tolerance. The MISS consists of 12 items covering four domains: stomachache, nausea, vomiting, and behavioural symptoms. The latter covers whether the child is restless, irritable or cries when low-dose MTX is administered or refuses low-dose MTX. The MISS addresses whether stomachache or nausea occur before or after MTX administration or when thinking of MTX; and whether vomiting occurs before or after MTX administration. For each item the severity is graded: no complaints (0 points), mild complaints (1 point), moderate complaints (2 points) or severe complaints (3 points). A total score is calculated summing up all the items: ranging from 0 to 36. To be categorised as MTX intolerant, the total score must be 6 or above (the cut-off score), with at least 1 point in an anticipatory and/or associative and/or behavioural symptom. The Danish version of the MISS was translated from an English original provided by the authors of the development paper [6]. The translation was performed by three of the authors of this manuscript: a medical doctor, a senior physician and professor in paediatric rheumatology, and a professor in psychology. All have Danish as their first language. A professional translator revised the final translation (S1 File).

The PGA_mtx_ consisted of a VAS-score (0-100mm) with the endpoints “no MTX intolerance” and “severe MTX intolerance” and was performed in line with the MISS development paper [6] and hence did not include laboratory tests of liver enzymes.

The two questions on MTX treatment compliance were: how often the day for low-dose MTX administration had been changed due to nausea during the last three months prior to enrolment in this study? How often the low-dose MTX administration had been skipped due to nausea during the last three months prior to enrolment in this study? The response categories were: 0 times; 1–3 times; 4–6 times; 7–9 times or 10–12 times.

### Statistical analyses

The statistical analyses used to evaluate the internal consistency of the Danish version of the MISS were Cronbach alpha (including Cronbach alpha if an item is deleted), corrected item-total correlations (the item correlated to the rest of the scale) and inter-domain correlations. Data on demographics, low-dose MTX specific elements and MTX intolerance measurements were analysed using Wilcoxon rank-sum test, Spearman’s rho (with degrees of freedom) and chi-square test/Fisher’s exact test. All statistical analyses were performed in STATA-13.

## Results

**Fig 1:**
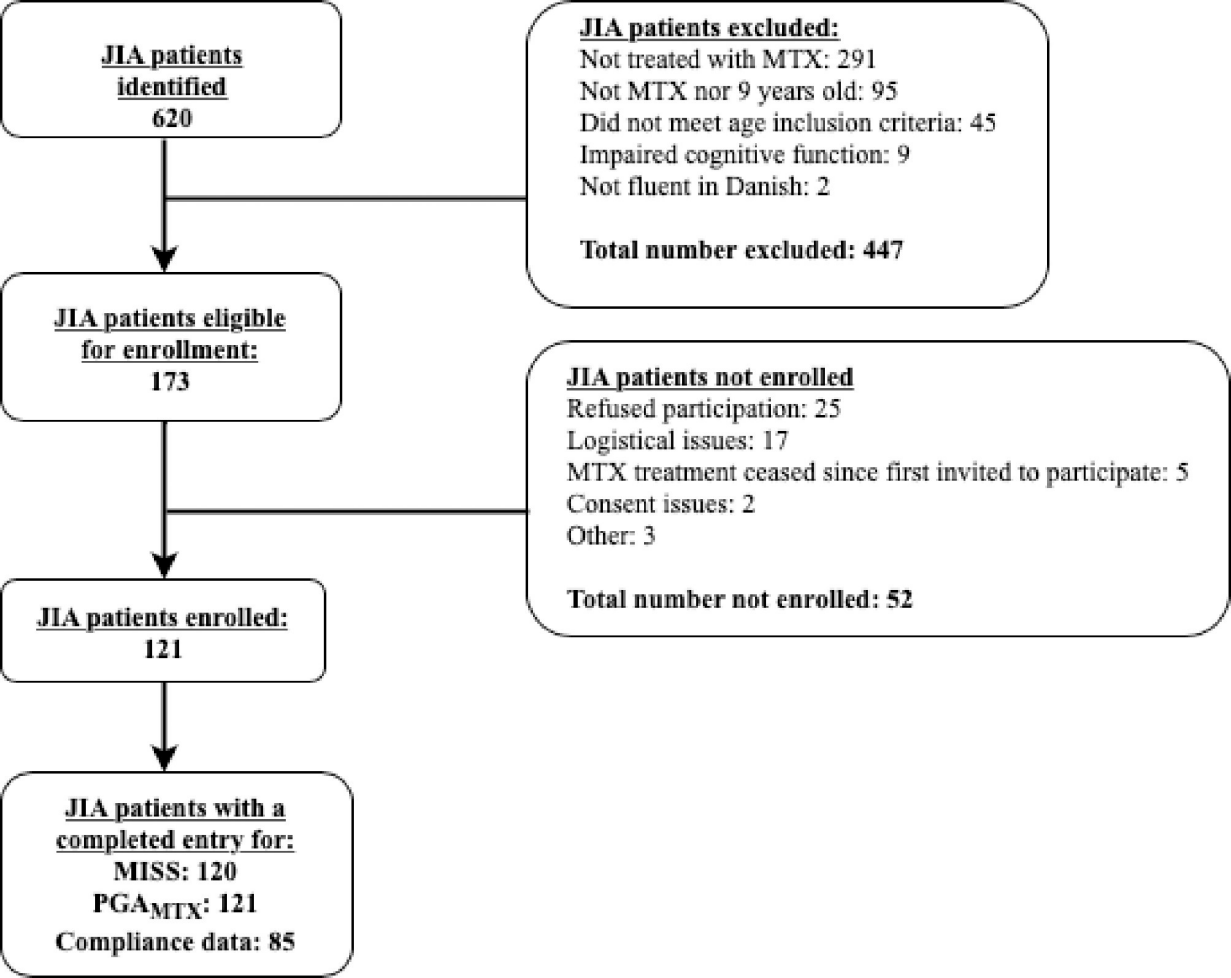
The juvenile idiopathic arthritis study population. Flowchart showing the enrolment and exclusion of patients within the juvenile idiopathic arthritis (JIA) study population. MTX; low-dose methotrexate. MISS; Methotrexate intolerance severity score. PGA_mtx_; Physician’s global assessment of MTX intolerance.

**Fig 2:**
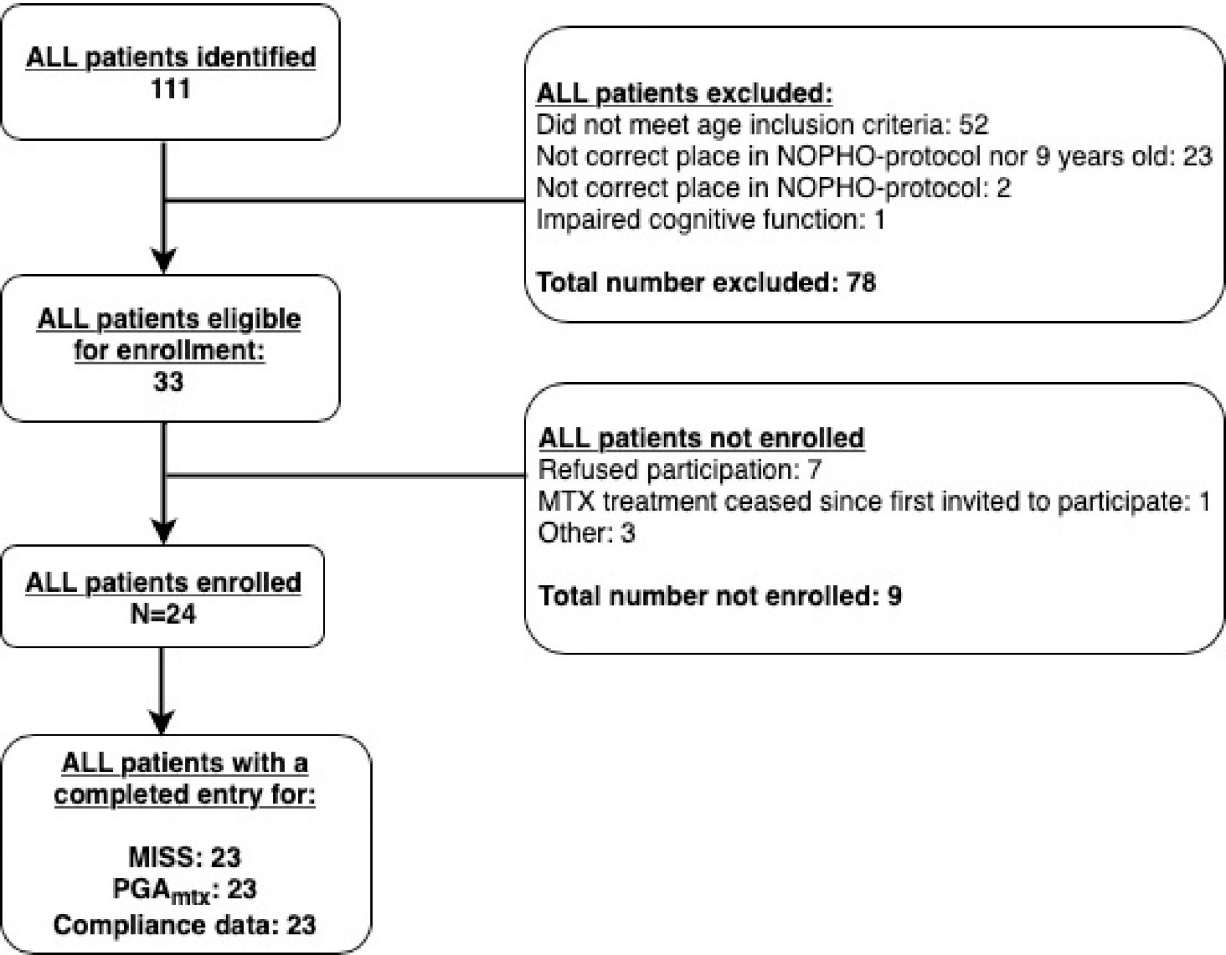
The acute lymphoblastic leukaemia study population. Flowchart showing the enrolment and exclusion of patients within the acute lymphoblastic leukaemia (ALL) study population. NOPHO = Nordic Society of Paediatric Haematology and Oncology. MTX = low-dose methotrexate; MISS; Methotrexate intolerance severity score. PGA_mtx_; Physician’s global assessment of MTX intolerance.

The study population included 120 children with JIA and 23 with ALL. Figs 1 and 2 show the details of exclusion and non-enrolment within the two disease groups. In one patient from each group data was missing due to the parents never completing the MISS, despite having given written consent to participation. These two patients have been excluded from the statistical analyses. For the JIA group, data on compliance was available for 85 patients due to the questions being added after the enrolment had started.

### Demographics and low-dose MTX details

The median age in the two disease groups did not differ significantly. In the JIA group there were twice as many girls as boys, whereas in the ALL group the gender distribution was equal (Table 1). The median MTX dose was significantly higher in the ALL group than in the JIA group. Within the JIA group there was no difference in the median MTX dose between the MTX_sc_ subgroup and the MTX_O_ subgroup (Table 1). The median duration of receiving low-dose MTX was twice as long in the JIA group as in the ALL group (Table 1). At enrolment, the entire JIA group had folic acid supplement prescribed (5mg the day after administration of low-dose MTX), but none in the ALL group. Similar proportions in the two disease groups had antiemetic medicine prescribed at enrolment (Table 1). Within the JIA group, a significantly larger proportion of children had antiemetic medicine prescribed in the MTX_intolerant_ subgroup compared to the MTX_tolerant_ subgroup. In the ALL group, there was no difference in the proportion of children with antiemetic medicine prescribed in the MTX_intolerant_ subgroup compared to the MTX_tolerant_ subgroup.

**Table 1:** Demographics and methotrexate intolerance.

Patient group	JIA	ALL	p-value
**Number of patients**	120	23	-
**Girls: Boys, n (% Females)**	81:39 (68)	10:13 (44)	-
**Age at enrolment, years (range)**	13.3 (9.1–17.4)	11.8 (9.6–18.6)	0.12
**MTX**_**O**_**: MTX**_**SC**_**, n**	45:75	23:0	-
**If currently MTX**_**SC**_**: number ever treated with MTX**_**O**_ **, n (%)**	48/75 (64)	0	-
**If currently MTX**_**O**_**: number ever treated with MTX**_**SC**_**, n (%)**	14/45 (31)	0	-
**MTX dose (mg/m**^**2**^**/week)**	9.8 (9.0–11.0)	14.4 (10.4–20.0)	0.0002
**MTX**_**o**_	9.6 (9.0–10.7)^c^	-	^c^0.81
**MTX**_**SC**_	9.8 (8.7–11.1)	-	**-**
**MTX**_**intolerant**_^**a**^	9.6 (8.6–11.0)^c^	16.3 (14.7–18.4)^d^	^c^0.18; ^d^0.47
**MTX**_**tolerant**_	10.1(9.4–10.9)	12.7 (9.0–21.0)	**-**
**MTX treatment duration**^**b**^ **(days)**	338 (142–765)	115 (71–266)	0.0006
**MTX**_**intolerant**_^**a**^	370 (143–766)^c^	300 (191–354)^d^	^c^0.44; ^d^0.06
**MTX**_**tolerant**_	264 (141–738)	99 (64–211)	-
**Antiemetic medicine prescribed at enrolment, n (%)**	41/120 (34)	8/22^e^ (36)	-
**MTX**_**intolerant**_^**a**^	33/73^c^	1/3^d^	^c^**χ**^**2**^ = 10.1; p = 0.001
**MTX**_**tolerant**_	8/47	7/19	^d^1.00
**PGA**_**mtx**_ **(0-100mm)**	18 (0–37)	3 (0–14)	0.08
**MISS total score (0–36)**	8 (3–14)	1 (0–3)	<0.0001
**MTX**_**O**_	6 (1–12)^c^	-	^c^0.044
**MTX**_**SC**_	9 (4–14)	-	-
**MTX**_**intolerant**_^**a**^ **patients, n (%)**	73 (61)	4 (17)	<0.001
**MTX**_**O**_**: MTX**_**SC**_**, n**	23: 50^c^	-	^c^**χ**^**2**^ = 2.86; p = 0.09
**Girls: boys**	50:23^c^	2:2^d^	^c^0.84; ^d^1.00

Data on demographic factors, the low-dose methotrexate treatment, prescribed antiemetic medicine, and the measures of methotrexate intolerance for both the juvenile idiopathic arthritis group (n = 120) and the acute lymphoblastic leukaemia group (n = 23). Values are expressed as median (IQR) unless otherwise stated. JIA; Juvenile idiopathic arthritis. ALL; Acute lymphoblastic leukaemia. MTX_SC;_ Subcutaneous route of administration of low-dose MTX. MTX_o_; Oral route of administration of low-dose MTX. PGA_mtx;_ Physician’s global assessment of MTX intolerance. MISS; The methotrexate intolerance severity score.

^a^ MTX_intolerant_ when the child’s total score of the methotrexate intolerance severity score (MISS) ≥6, with at least 1 point in an anticipatory and/or associative and/or behavioral symptom.

^b^ Duration of treatment with low-dose MTX prior to enrolment

^c^ Statistical comparison within the JIA group

^d^ Statistical comparison within the ALL group

^e^ Missing information on prescription of antiemetic medicine for 1 patient with ALL

In the JIA group, 62 (52%) of the children had previously changed the route of administration of low-dose MTX, primarily from oral to subcutaneous administration. None in the ALL group had previously changed the route of administration of low-dose MTX (Table 1). During the last 3 months prior to enrolment, none of the children with ALL had changed the weekly day for receiving low-dose MTX due to nausea, nor had they skipped any doses of MTX due to nausea (Table 2). In the JIA group 30 (35%) of the 85 children asked had changed the weekly day for receiving low-dose MTX due to nausea; and nine (11%) had skipped at least one administration of low-dose MTX due to nausea (Table 2).

**Table 2:** Low-dose MTX treatment compliance.

Patient group	JIA^a^		ALL
	MTX_O_	MTX_SC_	MTX_O_
**Number of times the patient has changed the day of low-dose MTX administration due to nausea:**			
**Never**	26	29	23
**1–3 times**	6	13	0
**4–6 times**	2	6	0
**7–9 times**	1	1	0
**10–12 times**	0	1	0
**Number of times the patient has skipped an administration of low-dose MTX due to nausea:**			
**Never**	32	44	23
**1–3 times**	2	5	0
**4–6 times**	1	1	0
**7–9 times**	0	0	0
**10–12 times**	0	0	0

Data on reported low-dose methotrexate treatment compliance during the last 3 months prior to enrolment within the juvenile idiopathic arthritis (JIA) group and the acute lymphoblastic leukaemia (ALL) group. MTX_SC;_ Subcutaneous route of administration of low-dose MTX. MTX_o_; Oral route of administration of low-dose MTX

^a^ Data available on 85 patients with JIA as the questions were introduced after the commencement of the enrolment period.

### Reliability of the Danish version of the MISS

For the JIA group there was a good internal consistency (Cronbach alpha = 0.87) and no single item deletion led to significant increase in Cronbach alpha. The corrected item-total correlations were all positive, and ranged from 0.3 to 0.7 (Table 3). The inter-domain correlations ranged from 0.3 to 0.6 (Table 4).

**Table 3:** The internal consistency of the methotrexate intolerance severity score–the juvenile idiopathic arthritis group.

Item	No complaints (0 points)	Mild complaints (1 point)	Moderate complaints (2 points)	Severe complaints (3 points)	Mean (95% CI)	Corrected item-total correlation	Cronbach alpha if item deleted
**1: stomachache after MTX**	53 (44%)	30 (25%)	30 (25%)	7 (6%)	0.9 (0.75–1.10)	0.57	0.86
**2: stomachache before MTX**	76 (63%)	24 (20%)	15 (13%)	5 (4%)	0.58 (0.42–0.73)	0.67	0.86
**3: stomachache when thinking of MTX**	67 (56%)	19 (16%)	19 (16%)	15 (13%)	0.85 (0.65–1.05)	0.67	0.86
**4: nauseous after MTX**	32(27%)	27(23%)	36 (30%)	25 (21%)	1.45 (1.25–1.65)	0.58	0.86
**5: nauseous before MTX**	65 (54%)	29 (24%)	15 (13%)	11 (9%)	0.77 (0.59–0.95)	0.52	0.87
**6: nauseous when thinking of MTX**	51(43%)	28 (23%)	20 (17%)	21(18%)	1.09 (0.89–1.30)	0.65	0.86
**7: vomit after MTX**	92 (77%)	10 (8%)	9 (7.5%)	9 (7.5%)	0.46 (0.29–0.63)	0.57	0.86
**8: vomit before MTX**	113 (94%)	5 (4%)	1 (1%)	1 (1%)	0.08 (0.01–0.15)	0.32	0.88
**9: restless when taking MTX**	66 (55%)	19 (16%)	22 (18%)	13 (11%)	0.85 (0.66–1.04)	0.61	0.86
**10: cries when taking MTX**	86 (72%)	15 (13%)	12 (10%)	7 (6%)	0.50 (0.34–0.66)	0.50	0.87
**11: irritable when taking MTX**	55 (46%)	29 (24%)	21 (18%)	15 (13%)	0.97 (0.77–1.16)	0.60	0.86
**12: refuses to take MTX**	88 (73%)	19 (16%)	7 (6%)	6 (5%)	0.43 (0.28–0.57)	0.50	0.87

The analysis of internal consistency of the Danish version of the methotrexate intolerance severity score (MISS) for the juvenile idiopathic arthritis group (n = 120), including the Cronbach alpha if an item is deleted and the corrected item-total.

MTX; low-dose methotrexate

**Table 4:** Inter-domain correlations of the MISS–the juvenile idiopathic arthritis group.

JIA	Stomachache	Nausea	Vomiting	Behaviour
**Stomachache**	1.0			
**Nausea**	0.5675	1.0		
**Vomiting**	0.5067	0.5360	1.0	
**Behaviour**	0.4667	0.3666	0.3111	1.0

The results of the inter-domain correlations of the four domains of the methotrexate intolerance severity score (MISS) for the juvenile idiopathic arthritis group (JIA) group (n = 120). All correlations are Spearman’s rho.

**Fig 3:**
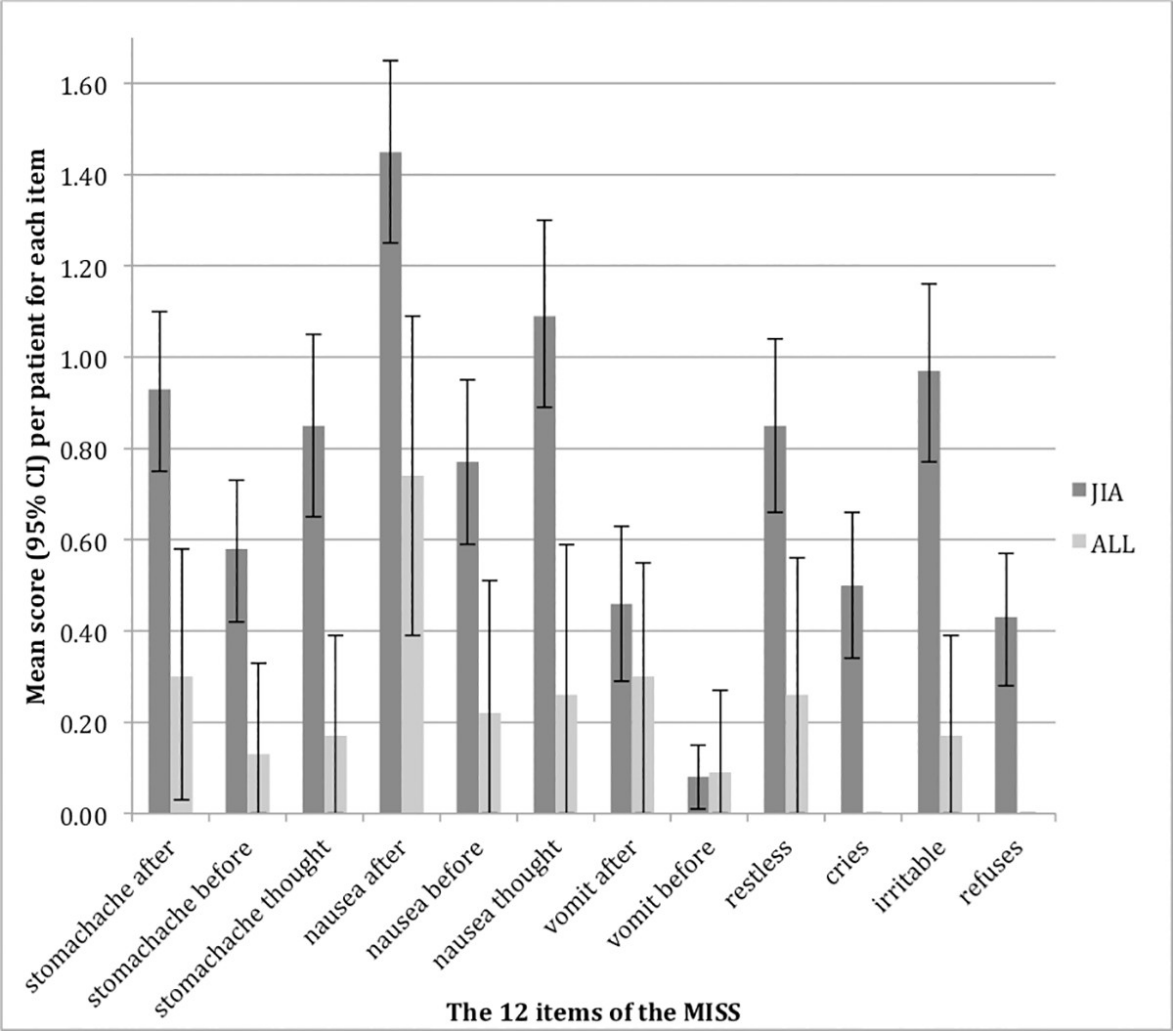
The methotrexate intolerance severity score–the juvenile idiopathic arthritis group and the acute lymphoblastic leukaemia group. Bar chart illustrating the mean scores (with 95% confidence interval) for each of the 12 items of the methotrexate intolerance severity score (MISS) for the juvenile idiopathic arthritis (JIA) group (n = 120) and the acute lymphoblastic leukaemia (ALL) group (n = 23).

For the ALL group, none of the children had complaints for the items: “refusal of low-dose MTX” and “cries when low-dose MTX is administered” (Fig 3). Hence the high Cronbach alpha of 0.88 was based on 10 items. Deletion of the remaining two behaviour items caused the Cronbach alpha to increase to 0.90. The corrected item-total correlations were positive and ranged from 0.6 to 0.9, if disregarding the remaining two behaviour items (r = -0.02 and r = 0.26) (Table 5). The inter-domain correlations ranged from 0.6 to 0.9, apart from the behaviour domain, which correlated poorly (r≤0.27) to all other domains (Table 6).

**Table 5:** The internal consistency of the methotrexate intolerance severity score–the acute lymphoblastic leukaemia group.

Item	No complaints (0 points)	Mild complaints (1 point)	Moderate complaints (2 points)	Severe complaints (3 points)	Mean (95% CI)	Corrected item-total correlation	Cronbach alpha if item deleted
**1: stomachache after MTX**	18 (78%)	3 (13%)	2 (9%)	0	0.30 (0.03–0.58)	0.75	0.86
**2: stomachache before MTX**	21 (91%)	1 (4%)	1 (4%)	0	0.13 (0–0.33)	0.67	0.87
**3: stomachache when thinking of MTX**	20 (87%)	2 (9%)	1 (4%)	0	0.17 (0–0.39)	0.87	0.85
**4: nauseous after MTX**	11 (48%)	7 (30%)	5 (22%)	0	0.74 (0.39–1.09)	0.62	0.87
**5: nauseous before MTX**	20 (87%)	2 (9%)	0	1 (4%)	0.22 (0–0.51)	0.80	0.85
**6: nauseous when thinking of MTX**	20 (87%)	1 (4%)	1 (4%)	1 (4%)	0.26 (0–0.59)	0.88	0.84
**7: vomit after MTX**	17 (74%)	5 (22%)	1 (4%)	0	0.30 (0.06–0.55)	0.63	0.87
**8: vomit before MTX**	22 (96%)	0	1 (4%)	0	0.09 (0–0.27)	0.77	0.86
**9: restless when taking MTX**	19 (83%)	3 (13%)	0	1 (4%)	0.26 (0–0.56)	0.26	0.90
**10: cries when taking MTX**	23 (100%)	0	0	0	0	-	-
**11: irritable when taking MTX**	20 (87%)	2 (9%)	1 (4%)	0	0.17 (0–0.39)	-0.02	0.90
**12: refuses to take MTX**	23 (100%)	0	0	0	0	-	-

The analysis of internal consistency of the Danish version of the methotrexate intolerance severity score (MISS) for the acute lymphoblastic leukaemia (ALL) group (n = 23), including the Cronbach alpha if an item is deleted and the corrected item-total.

MTX; low-dose methotrexate

**Table 6:** Inter-domain correlations of the MISS–the acute lymphoblastic leukaemia group.

ALL	Stomachache	Nausea	Vomiting	Behaviour
**Stomachache**	1.0			
**Nausea**	0.8950	1.0		
**Vomiting**	0.5860	0.7504	1.0	
**Behaviour**	0.0305	0.2732	0.1666	1.0

The inter-domain correlations of the four domains of the methotrexate intolerance severity score (MISS) for the acute lymphoblastic leukaemia (ALL) group (n = 23). All correlations are Spearman’s rho.

### Methotrexate intolerance

The median MISS score was significantly higher in the JIA group than in the ALL group; and the JIA group had a significantly larger proportion of MTX intolerant children than the ALL group (Table 1). For 17 (14%) of the children with JIA and 9 (39%) of the children with ALL, parents reported that their child had no complaints to low-dose MTX (MISS = 0). Within the JIA group, there was no significant difference between the proportion of MTX intolerant children in the MTX_SC_ subgroup and the MTX_O_ subgroup (Table 1). The median MISS score was above the cut-off score in both the MTX_SC_ subgroup and the MTX_O_ subgroup—it was slightly higher in the MTX_SC_ subgroup compared to the MTX_O_ subgroup (Table 1).

No significant difference was found between the two disease groups’ median PGA_mtx_ (Table 1). The PGA_mtx_ showed a high correlation with the total MISS and out of the four domains of the MISS, the PGA_mtx_ had the highest correlation with the nausea domain (Table 7).

**Table 7:** Methotrexate-intolerance: Physician and parent assessment.

Patient group	JIA	ALL
**PGA**_**mtx**_ **correlated to:**		
**MISS**_**total score**_	r_s_(118) = 0.5317; p <0.0001	r_s_(21) = 0.5845; p = 0.0034
**MISS**_**stomachache domain**_	r_s_(118) = 0.3046; p = 0.0007	r_s_(21) = 0.3073; p = 0.15
**MISS**_**nausea domain**_	r_s_(118) = 0.5584; p <0.0001	r_s_(21) = 0.5585; p = 0.0056
**MISS**_**vomit domain**_	r_s_(118) = 0.3288; p = 0.0002	r_s_(21) = 0.2987; p = 0.17
**MISS**_**behavioral domain**_	r_s_(118) = 0.3969; p <0.0001	r_s_(21) = 0.5127; p = 0.0124

The correlations between the physician’s global assessment of the patients’ current level of methotrexate intolerance (PGA_mtx_) and the methotrexate intolerance severity score (MISS)–the total score and the scores for the four domains–for the juvenile idiopathic arthritis (JIA) group (n = 120) and the acute lymphoblastic leukemia (ALL) group (n = 23). r_s_; Spearman’s Rho (degrees of freedom)

The parents of the patients with JIA reported that their child most often experienced nausea after MTX administration (73%), nausea when thinking of MTX (58%), stomachache after MTX administration (56%) and that their child was irritable when MTX was administered (54%). Nearly half of the patients with JIA experienced nausea before MTX (46%), were restless when MTX was administered (45%) or experienced stomachache when thinking of MTX (44%) (Fig 3, Table 3).

For the ALL group, 52% of the parents reported that their child had mild to moderate complaints of nausea after low-dose MTX administration. For the remaining items, the majority of the parents (74–96%) reported that their child had “no complaints” (Fig 3, Table 5). Very few (4%) of the patients with ALL experienced severe complaints on an item and only on items covering an anticipatory or behavioural symptom (Table 5).

Within neither the JIA group nor the ALL group was a significant correlation found between the total MISS scores and age (JIA group: r_s_(118) = -0.13, p = 0.16; ALL group: r_s_(21) = 0.05; p = 0.81), and no significant difference was found between the number of girls and boys being MTX intolerant (Table 1). No significant correlation was found between the total MISS scores and the MTX doses in either of the disease groups (JIA group: r_s_(118) = -0.17, p = 0.06; ALL group: r_s_(21) = 0.34; p = 0.11), and no significant difference was found between the median MTX dose in the MTX_tolerant_ subgroup and the MTX_intolerant_ subgroup in either of the disease groups (Table 1). In both disease groups no significant correlation was found between the total MISS scores and the MTX treatment duration (JIA group: r_s_(118) = 0.046, p = 0.62; ALL group: r_s_(21) = 0.23; p = 0.30), and no significant difference was found between the median MTX treatment duration in the MTX_tolerant_ subgroup and the MTX_intolerant_ subgroup in either disease group (Table 1).

## Discussion

This Danish version of the MISS had a high internal consistency in the JIA group, indicating the Danish translation of the MISS was satisfactory and supporting other studies in the MISS being easy to adapt to a new language [15–17]. For the ALL group the analysis of internal consistency questioned whether the behaviour domain fits properly with the remaining MISS. Very few of the parents of the children with ALL reported that their child had complaints within the behaviour domain and none within the items “refusal of low-dose MTX” and “cries when low-dose MTX is administered”. One could thus argue for a separate MISS scale for the ALL group, where the behaviour domain is excluded. However, in daily practice it is preferable to use a uniform scale across different diseases. Furthermore, the behaviour domain proved clinically relevant for the patients with ALL actually affected within the domain. Moreover, if the ALL study population had been larger in size it is plausible that more patients would have had complaints within the behaviour domain. The numeric value of the MISS cut-off score has been debated [6,15]. Hence, when using the MISS it may be more clinically relevant to focus on the ability of the MISS to uncover which aspects of MTX intolerance an individual child is affected by and use the total score for monitoring the patient. Used in this manner the MISS may also be applicable within leukaemia care.

A larger proportion of the JIA group than of the ALL group experienced the low-dose MTX related adverse effects specified by the MISS and to a higher degree. This difference was not found when assessed by physicians. The differing results may be explained by the assessment of MTX intolerance only composing one of many disease aspects physicians considered when seeing patients in the outpatient clinics, compared to parents focusing on MTX intolerance during the enrolment into this project. The correlations of the PGA_mtx_ to the MISS illustrated that the two measures differ slightly in their focus, possibly also explaining the differing results.

When assessed by the parents, patients with JIA most often experienced mild to severe nausea after MTX administration, but anticipatory, associative, and behavioural symptoms contributed significantly to the overall MTX intolerance within the JIA group. For the patients with ALL the MTX intolerance issues were primarily nausea after MTX administration and only in a mild to moderate degree. When patients with ALL did experience severe complaints, it was in relation to anticipatory and associative nausea and a behavioural complaint.

Our finding of 61% of the JIA group being categorised as MTX intolerant is surprisingly high compared to previous studies where approximately 25% were MTX intolerant at selected time points within the study period [16,18] and 42–50% if looking at the proportion of JIA patients scored MTX intolerant at any one point during the entire study period [6,16–18].

The JIA group in our study had been treated with low-dose MTX for a long period of time with a median duration of 338 days. This may contribute to the high number of MTX intolerant children in our JIA group. Especially, when studies have shown that MTX intolerance develops within the first year of low-dose MTX treatment and stays rather stable over time [7,16,18]. However, the duration of low-dose MTX treatment in our JIA group is comparable to the study by Scheuern et al. [7] finding a lower proportion of MTX intolerant patients with JIA. Additionally, in our study the median duration of low-dose MTX treatment was the same in the JIA subgroup of MTX_tolerant_ patients as in the JIA subgroup of MTX_intolerant_ patients.

In the study by Bulatovic et al. [6] more patients were rated MTX intolerant in the subgroup treated with subcutaneous low-dose MTX compared to the subgroup treated with oral low-dose MTX. Hence, the high proportion of our JIA group receiving low-dose MTX subcutaneously (62.5%) may contribute to the higher proportion of MTX intolerant children with JIA in our study. However, as argued in the study by Patil et al. [19] changing the route of administration is a known attempted countermeasure to MTX intolerance [15,16,20,21]. We did indeed see that 64% of the children with JIA treated with subcutaneous low-dose MTX at enrolment had previously received low-dose MTX orally. Due to our cross-sectional design we can only speculate that MTX intolerance was developed during oral MTX treatment and may be the cause for the change to subcutaneous MTX treatment. It should further be noted, that in the study by Bulatovic et al. [6] the median MISS score was exactly the same in the subgroup treated with oral low-dose MTX as in the subgroup treated with subcutaneous low-dose MTX. Furthermore, in the study by Franova et al. [16] an even higher proportion (82%) of children with JIA received low-dose MTX subcutaneously and still only 30% of the patients with JIA were evaluated MTX intolerant after 12 months of treatment. Additionally, in line with our findings, the studies by Scheuern et al. [7] and van Dijkhuizen et al. [18] found the proportion of children with JIA receiving subcutaneous low-dose MTX was the same in the MTX_intolerant_ group as in the MTX_tolerant_ group.

Our results surprisingly show that children with ALL in maintenance treatment with low-dose MTX (after completed reinductions) had a low level of MTX intolerance and only few patients were affected by anticipatory and associative symptoms, when assessed by their parents. This is remarkable considering that anticipatory and associative nausea and the concept of a conditioned response are well known phenomena associated with the initial high-dose chemotherapy (including high-dose MTX) [22,23]. The majority of patients with ALL must somehow have reversed the conditioned response indicating that there are lessons to be learnt from the ALL group regarding the handling of MTX intolerance.

A limitation to our study is that the compliance results are retrospective with the risk of recall bias. However, there is no reason to expect one disease group to recall more poorly than the other. The difference in compliance between the ALL group and the JIA group could be due to the higher level of MTX intolerance in the JIA group, but it is more likely due to the difference in disease types–with ALL being a malignancy. Furthermore, this is the only retrospective element of this study.

Another limitation is that children below the age of 9 years were not included. However, as the parents completed the questionnaire in our study we believe this Danish version of the MISS is applicable for use in younger children as well. It may be considered a limitation that the two disease groups have study populations of different sizes. The proportions of excluded patients and non-enrolled patients are similar in the JIA group and the ALL group, thus it seems reasonable to assume that the difference in size of the two study populations reflects the difference in prevalence and incidence of the two diseases at the investigated age interval.

Our results uncover that MTX intolerance is diverse and varies both within and between disease types. Hence the management of MTX intolerance needs to the tailored to the individual patient, supporting the recommendation by Franova et al. [16]. Antiemetic medicine seems as an evident intervention to minimise the nausea after low-dose MTX administration. It has even been proposed that antiemetic medicine should be introduced very early in order to avoid the development of a conditioned response–i.e. anticipatory nausea [23,24]. Supported by Dupont-Lucas et al. [24] who found that children with inflammatory bowel disease receiving antiemetic medicine secondarily, compared to prophylactic, were more likely to be MTX intolerant. Once anticipatory and associative symptoms are present a psychological intervention–e.g. cognitive behavioural therapy–is a possible approach [23,25]. The psychological intervention eye movement desensitisation and reprocessing has recently been described to have short-term efficacy for minimising MTX intolerance [26].

In conclusion, the level of MTX intolerance was higher and more attributed to anticipatory, associative, and behavioural symptoms in the JIA group compared to the ALL group. We regard the MISS as a useful tool within JIA to uncover whether MTX intolerance is present, to elucidate which items are affected in the individual patient and hence how best to intervene. The MISS may be applicable within leukaemia care, but further studies are warranted to support our findings and possibly investigate psychological factors as contributors to the difference between the two disease groups.

## Supporting information

S1 File Danish MISS. A Danish translation of the methotrexate intolerance severity score (MISS).
(PDF)

S2 File Minimal data set. (XLS)

## References

[1] Giannini EH, Brewer EJ, Kuzmina N, Shaikov A, Maximov A, Vorontsov I, et al Methotrexate in resistant juvenile rheumatoid arthritis. Results of the U.S.A.-U.S.S.R. double-blind, placebo-controlled trial. The Pediatric Rheumatology Collaborative Study Group and The Cooperative Children's Study Group. N Engl J Med 1992 4 16;326(16):1043–1049. 10.1056/NEJM199204163261602 1549149

[2] Hashkes PJ, Laxer RM. Medical treatment of juvenile idiopathic arthritis. JAMA 2005 10 5;294(13):1671–1684. 16204667 10.1001/jama.294.13.1671

[3] Beukelman T, Patkar NM, Saag KG, Tolleson-Rinehart S, Cron RQ, DeWitt EM, et al 2011 American College of Rheumatology recommendations for the treatment of juvenile idiopathic arthritis: initiation and safety monitoring of therapeutic agents for the treatment of arthritis and systemic features. Arthritis Care Res (Hoboken) 2011 4;63(4):465–482. 21452260 10.1002/acr.20460 PMC3222233

[4] Toft N, Birgens H, Abrahamsson J, Bernell P, Griskevicius L, Hallbook H, et al Risk group assignment differs for children and adults 1–45 yr with acute lymphoblastic leukemia treated by the NOPHO ALL-2008 protocol. Eur J Haematol 2013 5;90(5):404–412. 10.1111/ejh.12097 23461707

[5] van der Meer A, Wulffraat NM, Prakken BJ, Gijsbers B, Rademaker CM, Sinnema G. Psychological side effects of MTX treatment in juvenile idiopathic arthritis: a pilot study. Clin Exp Rheumatol 2007 May-Jun;25(3):480–485. 17631750

[6] Bulatovic M, Heijstek MW, Verkaaik M, van Dijkhuizen EH, Armbrust W, Hoppenreijs EP, et al High prevalence of methotrexate intolerance in juvenile idiopathic arthritis: development and validation of a methotrexate intolerance severity score. Arthritis Rheum 2011 7;63(7):2007–2013. 10.1002/art.30367 21437879

[7] Scheuern A, Tyrrell PN, Haas JP, Hugle B. Countermeasures against methotrexate intolerance in juvenile idiopathic arthritis instituted by parents show no effect. Rheumatology (Oxford) 2017 6 1;56(6):901–906. 28122960 10.1093/rheumatology/kew507

[8] Levinsen M, Rosthoj S, Nygaard U, Heldrup J, Harila-Saari A, Jonsson OG, et al Myelotoxicity after high-dose methotrexate in childhood acute leukemia is influenced by 6-mercaptopurine dosing but not by intermediate thiopurine methyltransferase activity. Cancer Chemother Pharmacol 2015 1;75(1):59–66. 10.1007/s00280-014-2613-7 25347948 PMC4446052

[9] Lopez-Lopez E, Gutierrez-Camino A, Bilbao-Aldaiturriaga N, Pombar-Gomez M, Martin-Guerrero I, Garcia-Orad A. Pharmacogenetics of childhood acute lymphoblastic leukemia. Pharmacogenomics 2014 7;15(10):1383–1398. 10.2217/pgs.14.106 25155938

[10] Pui CH, Schrappe M, Ribeiro RC, Niemeyer CM. Childhood and adolescent lymphoid and myeloid leukemia. Hematology Am Soc Hematol Educ Program 2004:118–145. 10.1182/asheducation-2004.1.118 15561680

[11] Brunner HI, Johnson AL, Barron AC, Passo MH, Griffin TA, Graham TB, et al Gastrointestinal symptoms and their association with health-related quality of life of children with juvenile rheumatoid arthritis: validation of a gastrointestinal symptom questionnaire. J Clin Rheumatol 2005 8;11(4):194–204. 16357756 10.1097/01.rhu.0000173616.81928.44

[12] Petty RE, Southwood TR, Manners P, Baum J, Glass DN, Goldenberg J, et al International League of Associations for Rheumatology classification of juvenile idiopathic arthritis: second revision, Edmonton, 2001. J Rheumatol 2004 2;31(2):390–392. 14760812

[13] Toft N, Birgens H, Abrahamsson J, Griskevicius L, Hallbook H, Heyman M, et al Results of NOPHO ALL2008 treatment for patients aged 1–45 years with acute lymphoblastic leukemia. Leukemia 2017 8 18. 10.1038/leu.2017.265 28819280

[14] Harris PA, Taylor R, Thielke R, Payne J, Gonzalez N, Conde JG. Research electronic data capture (REDCap)—a metadata-driven methodology and workflow process for providing translational research informatics support. J Biomed Inform 2009 4;42(2):377–381. 10.1016/j.jbi.2008.08.010 18929686 PMC2700030

[15] Chausset A, Fargeix T, Pereira B, Echaubard S, Duquesne A, Desjonqueres M, et al MISS questionnaire in French version: a good tool for children and parents to assess methotrexate intolerance. Clin Rheumatol 2017 6;36(6):1281–1288. 10.1007/s10067-017-3638-1 28477218

[16] Franova J, Fingerhutova S, Kobrova K, Srp R, Nemcova D, Hoza J, et al Methotrexate efficacy, but not its intolerance, is associated with the dose and route of administration. Pediatr Rheumatol Online J 2016 6 14;14(1):36-016-0099-z. 10.1186/s12969-016-0099-z PMC4908704 27301536

[17] Scheuern A, Fischer N, McDonald J, Brunner HI, Haas JP, Hugle B. Mutations in the MTHFR gene are not associated with Methotrexate intolerance in patients with juvenile idiopathic arthritis. Pediatr Rheumatol Online J 2016 2 29;14(1):11-016-0071-y. 10.1186/s12969-016-0071-y PMC4772529 26928923

[18] van Dijkhuizen EH, Bulatovic Calasan M, Pluijm SM, de Rotte MC, Vastert SJ, Kamphuis S, et al Prediction of methotrexate intolerance in juvenile idiopathic arthritis: a prospective, observational cohort study. Pediatr Rheumatol Online J 2015 2 18;13:5-015-0002-3. eCollection 2015. 10.1186/s12969-015-0002-3 PMC4349799 25745368

[19] Patil P, Parker RA, Rawcliffe C, Olaleye A, Moore S, Daly N, et al Methotrexate-induced nausea and vomiting in adolescent and young adult patients. Clin Rheumatol 2014 3;33(3):403–407. 10.1007/s10067-013-2389-x 24108504 PMC3937539

[20] Zuber Z, Turowska-Heydel D, Sobczyk M, Banach-Gornicka M, Rusnak K, Piszczek A, et al Methotrexate efficacy and tolerability after switching from oral to subcutaneous route of administration in juvenile idiopathic arthritis. Reumatologia 2016;54(1):19–23. 10.5114/reum.2016.58757 27407272 PMC4847326

[21] Alsufyani K, Ortiz-Alvarez O, Cabral DA, Tucker LB, Petty RE, Malleson PN. The role of subcutaneous administration of methotrexate in children with juvenile idiopathic arthritis who have failed oral methotrexate. J Rheumatol 2004;31(1):179–182. 14705239

[22] Dupuis LL, Roscoe JA, Olver I, Aapro M, Molassiotis A. 2016 updated MASCC/ESMO consensus recommendations: Anticipatory nausea and vomiting in children and adults receiving chemotherapy. Support Care Cancer 2017 1;25(1):317–321. 10.1007/s00520-016-3330-z 27510314

[23] Roscoe JA, Morrow GR, Aapro MS, Molassiotis A, Olver I. Anticipatory nausea and vomiting. Support Care Cancer 2011 10;19(10):1533–1538. 10.1007/s00520-010-0980-0 20803345 PMC3136579

[24] Dupont-Lucas C, Grandjean-Blanchet C, Leduc B, Tripcovici M, Larocque C, Gervais F, et al Prevalence and Risk Factors for Symptoms of Methotrexate Intolerance in Pediatric Inflammatory Bowel Disease. Inflamm Bowel Dis 2017 2;23(2):298–303. 10.1097/MIB.0000000000001014 28107279

[25] Dupuis LL, Robinson PD, Boodhan S, Holdsworth M, Portwine C, Gibson P, et al Guideline for the prevention and treatment of anticipatory nausea and vomiting due to chemotherapy in pediatric cancer patients. Pediatr Blood Cancer 2014 8;61(8):1506–1512. 10.1002/pbc.25063 24753095

[26] Hofel L, Eppler B, Storf M, Schnobel-Muller E, Haas JP, Hugle B. Successful treatment of methotrexate intolerance in juvenile idiopathic arthritis using eye movement desensitization and reprocessing—treatment protocol and preliminary results. Pediatr Rheumatol Online J 2018 2 13;16(1):11-018-0228-y. 10.1186/s12969-018-0228-y PMC5809965 29433504

