# Supplementary material for: Increased methotrexate intolerance in juvenile idiopathic arthritis compared to acute lymphoblastic leukaemia in children
Source: PLoS One. 2019 Jul 11;14(7):e0219539. doi: 10.1371/journal.pone.0219539 (PMC6622540; doi:10.1371/journal.pone.0219539)
Supplement: S1 File — A Danish translation of the methotrexate intolerance severity score (MISS). (PDF) [file pone.0219539.s001.pdf]

ID-NR.________________

Methotrexat Intolerance Sværhedsgrads Score – MISS

	INGEN GENER	GENER (score 1-3 point)		
		Milde	Moderate	Svære
	0	1	2	3
**MAVEPINE**				
• Mit barn har mavepine efter indtag af methotrexat	☐	☐	☐	☐
• Mit barn har mavepine flere timer op til et døgn før indtag af methotrexat	☐	☐	☐	☐
• Mit barn har mavepine bare ved tanken om methotrexat	☐	☐	☐	☐
**KVALME**				
• Mit barn får kvalme efter indtag af methotrexat	☐	☐	☐	☐
• Mit barn får kvalme flere timer op til et døgn før indtag af methotrexat	☐	☐	☐	☐
• Mit barn får kvalme bare ved tanken om methotrexat	☐	☐	☐	☐
**OPKASTNING**				
• Mit barn kaster op efter indtag af methotrexat	☐	☐	☐	☐
• Mit barn kaster op flere timer til et døgn før indtag af methotrexat	☐	☐	☐	☐
**ADFÆRDSMÆSSIGE GENER**				
• Mit barn er uroligt, når det tager methotrexat	☐	☐	☐	☐
• Mit barn græder, når det tager methotrexat	☐	☐	☐	☐
• Mit barn er irritabelt, når det tager methotrexat	☐	☐	☐	☐
• Mit barn nægter at tage methotrexat	☐	☐	☐	☐
